# Ribosomal Protein L40e Fused With a Ubiquitin Moiety Is Essential for the Vegetative Growth, Morphological Homeostasis, Cell Cycle Progression, and Pathogenicity of *Cryptococcus neoformans*

**DOI:** 10.3389/fmicb.2020.570269

**Published:** 2020-11-05

**Authors:** Jingyu Zhao, Yali Yang, Yibin Fan, Jiu Yi, Chao Zhang, Zhongkai Gu, Weihua Pan, Julin Gu, Wanqing Liao, Wei Fang

**Affiliations:** ^1^Shanghai Key Laboratory of Molecular Medical Mycology, Department of Dermatology, Changzheng Hospital, Second Military Medical University, Shanghai, China; ^2^Department of Dermatology, Shanghai Eastern Hepatobiliary Surgery Hospital, Shanghai, China; ^3^Department of Dermatology, Shanghai Ninth People’s Hospital, School of Medicine, Shanghai Jiao Tong University, Shanghai, China; ^4^Department of Dermatology, Zhejiang Provincial People’s Hospital, People’s Hospital of Hangzhou Medical College, Hangzhou, China; ^5^The Institute of Biomedical Sciences, Fudan University, Shanghai, China

**Keywords:** *Cryptococcus neoformans*, ubiquitin, growth restriction, cellular morphology, virulence, immune evasion

## Abstract

Ubiquitin is a highly conserved protein required for various fundamental cellular processes in eukaryotes. Herein, we first report the contribution of the ubiquitin fusion protein Ubi1 (a ubiquitin monomer fused with the ribosome protein L40e, Rpl40e) in the growth and pathogenicity of *Cryptococcus neoformans*. *UBI1* deletion resulted in severe growth restriction of *C. neoformans*, whose growth rate was positively correlated with *UBI1* expression level. The growth defect of the *ubi1*Δ strain could be closely associated with its morphological abnormalities, such as its reduced ribosome particles. In addition, the *ubi1*Δ mutant also displayed increased cell ploidy, cell cycle arrest, and decreased intracellular survival inside macrophages. All these phenotypes were reversed by the reconstitution of the full-length *UBI1* gene or *RPL40a* domain. Mouse survival and fungal burden assays further revealed a severely attenuated pathogenicity for the *ubi1*Δ mutant, which is probably associated with its reduced stress tolerance and the induction of T-helper 1-type immune response. Taken together, Ubi1 is required for maintaining the vegetative growth, morphological homeostasis, cell cycle progression, and pathogenicity *in vivo* of *C. neoformans*. The pleiotropic roles of Ubi1 are dependent on the presence of Rpl40e and associated with its regulation of cryptococcal ribosome biogenesis.

## Introduction

*Cryptococcus neoformans* is an important invasive fungal pathogen, which could cause life-threatening meningoencephalitis in both the immunocompetent and immunocompromised population (Fang et al., 2015; Maziarz and Perfect, 2016). Globally, it is estimated that there are approximately 278,000 new cases of cryptococcal infections each year, which cause 181,000 deaths annually (Rajasingham et al., 2017). In mammalian hosts, phagocytic cell-based immunity is critical to controlling fungal infections (Casadevall et al., 2018). Reactive oxygen and nitrogen species generated by macrophages, neutrophils, and other phagocytic cells are implicated in killing *C. neoformans* and other fungal pathogens (Seider et al., 2010; May et al., 2016). However, *C. neoformans* can effectively sense and overcome the hostile living environment *in vivo* (such as limited nutrition, high temperature, oxidative stress, etc.) via a variety of virulence factors (Kronstad et al., 2012; Alspaugh, 2015; Zaragoza, 2019). For example, yeast cells escape phagocytosis by immune cells by producing a capsule, which can also promote the intracellular proliferation and extracellular dissemination of *C. neoformans* (O’Meara and Alspaugh, 2012). Melanin can effectively remove free radicals in the host and interfere with macrophage polarization, thereby enhancing the intracellular survival of *C. neoformans* (Zaragoza, 2019).

Ubiquitination is a reversible post-translational modification mechanism in eukaryotes, involved in a variety of biological processes such as ribosome biogenesis, transcriptional regulation, protein quality control, and signal transduction (Harper and Schulman, 2006). Over the last decade, people have gradually realized the potential roles of the ubiquitin system in the growth and pathogenicity of *C. neoformans*. The ubiquitin-proteasome inhibitor bortezomib significantly inhibits the production of the most critical pathogenic factor, the capsule, in *C. neoformans* (Geddes et al., 2016). Our previous studies revealed that the deubiquitinase Ubp5 is essential for the production of various pathogenic factors (such as high thermotolerance, melanin, and the capsule), stress responses, sexual reproduction, and pathogenicity in both *C. neoformans* and *Cryptococcus gattii* (Fang et al., 2012; Meng et al., 2016). Similarly, deletion of the ubiquitin ligase Fbp1 causes sexual reproduction defects, decreased stress tolerance, and attenuated virulence *in vivo* in *C. neoformans* (Liu et al., 2011; Liu and Xue, 2014). These studies suggest that the ubiquitin system is crucial for *C. neoformans* to adapt to the environmental niche of a mammalian host.

Ubiquitin is a core element in the ubiquitin-proteasome system, and its functions and molecular mechanisms in the growth and pathogenicity of fungal pathogens remain unclear. Ubiquitin usually exists as a polyubiquitin precursor (Ubi4) or a hybrid protein with a fused ribosomal protein, such as Rpl40e (Ubi1/2 precursors) or Rps31e (Ubi3 precursor) (Clague and Urbé, 2010). In *Saccharomyces cerevisiae*, the ribosomal protein moiety is required for 60S or 40S subunit production, subunit assembly, and subsequent translation (Finley et al., 1989; Fernández-Pevida et al., 2012). The ubiquitin moiety acts as a chaperone by facilitating efficient ribosome biogenesis (Lacombe et al., 2009; Martín-Villanueva et al., 2019, 2020). The *ubi1*Δ or *ubi2*Δ mutant shows a slow-growth phenotype while *UBI3* deletion or double deletions of *UBI1* and *UBI2* cause a lethal phenotype in *S. cerevisiae* (Finley et al., 1989). In *C. neoformans*, ubiquitin is mainly composed of two encoding genes: the *UBI1* gene (encoding a fusion protein of a ubiquitin monomer and Rpl40e) and the *UBI4* gene (encoding a polyubiquitin containing five ubiquitin repeats) (Spitzer and Spitzer, 1995). Here we first report the identification and characterization of the pleiotropic roles of *UBI1* in the growth and pathogenicity *in vivo* of *C. neoformans*.

## Experimental Procedures

### Strains, Plasmids, and Media

All the cryptococcal strains and plasmids used in this study are shown in Supplementary Table S1. The wild-type (WT) H99 strain was cultured on YPD agar medium (1% yeast extract, 2% peptone, and 2% dextrose) and different transformants were cultured on selective media (YPD plus 200 mg L^–1^ G418 or 100 mg L^–1^ nourseothricin). Condition media (YPD plus different concentrations of bathocuproinedisulfonic acid or cupric sulfate) were used to induce or suppress the expression of *UBI1* in promoter-reconstituted strains. Several media for phenotypic assays (such as capsule-inducing medium, DOPA/caffeic-acid medium, and Titan Cells Medium) and DMEM medium for the J774 macrophage killing assay were prepared as previously reported (Fang et al., 2012; Trevijano-Contador et al., 2018).

### Gene Disruption and Reconstitution

The *Ubi1* homolog of *C. neoformans* was identified by BLAST search of the serotype A (H99) genome database^1^. The *UBI1* gene (CNAG_00370) was disrupted by biolistic transformation, with the disruption cassette generated by overlapping PCR. Specifically, the upstream flanking region (982-bp) and downstream flanking region (859-bp) of the *UBI1* gene were amplified from the genomic DNA of H99 with the primer pairs ZJY001/ZJY002 and ZJY003/ZJY004, respectively. ZJY005 and M13F were used to amplify the neomycin phosphotransferase II (*NEO*, 2098-bp) cassette from the plasmid pJAF1. These PCR products were gel-purified and used as templates to generate a 3.9-kb *ubi1:NEO* deletion construct. The linearized construct was transformed into H99 using a gene gun (Toffaletti et al., 1993). Stable transformants were selected by G418 resistance and colony PCR, using appropriate primers. Deletion of *UBI1* was further confirmed by Southern blot hybridization; the NEO probe was generated by PCR with the primers NEO-F and NEO-R (data not shown).

To obtain the complete sequence of the *UBI1* gene, a 3.8-kb DNA fragment, containing an open reading frame (ORF), promoter, and terminator region, was PCR amplified from H99 genomic DNA, sequenced, and then cloned into the plasmid pCH233 using the In-Fusion^®^ EcoDry^TM^ Cloning System (Clontech, Palo Alto, CA, United States). The reconstructed plasmid pUBI1-NAT was linearized with *Bam*HI and transformed into the H99 *ubi1*Δ strain by biolistic transformation (Toffaletti et al., 1993). Positive colonies were screened on YPD agar plus nourseothricin. The reconstitution of *UBI1* was confirmed by diagnostic PCR (primers ZJY010 and ZJY011) and Southern blot hybridization using a NAT probe (primers NAT-F and NAT-R).

A copper-regulated mutant strain was constructed using the overlap PCR technique. A 982-bp fragment of the 5′ region of *UBI1* was identical to the upstream flanking region of the *ubi1:NEO* deletion construct. A 956-bp fragment, starting from the initiation codon of *UBI1*, was amplified with the primers ZJY012 and ZJY013. The 2664-bp Neo^R^-P*_*CTR*__4_* fragment was amplified from the plasmid pNEO-CTR4 with the primers ZJY014 and ZJY015. All the PCR products were gel-purified and then used as templates to generate a 4.6-kb overlap fragment with the primers ZJY001 and ZJY013, which was then transformed into H99 using a biolistic delivery system. Stable transformants were screened on YPD agar containing G418 and BCS. The copper-regulated *UBI1* mutants (P_CTR__4_*-UBI1*) were selected using colony PCR and confirmed by Southern blotting and real-time PCR.

To determine the contributions of the different Ubi1 domains to its biological functions, truncated *UBI1* gene fragments, the N-terminal domain (monoubiquitin, UB, 1–76) and the C-terminal domain (Ribosomal unit protein L40e, Rpl40e, 77-129), were cloned into the pCH233 vector. For the construction of the pUB-NAT vector, a 1624-bp fragment containing the *UBI1* promoter and monoubiquitin ORF was amplified from the H99 genomic DNA, using the primers ZJY008 and ZJY016, and infused with the *UBI1* terminator fragment (941-bp, primers ZJY017 and ZJY009) by overlap PCR. The truncated monoubiquitin gene fragment was inserted into digested plasmid pCH233 by *Xba*I using an infusion kit, as described above. The vector pRPL40a-NAT was constructed in a similar way: a 1478-bp fragment containing the RPL40a ORF and *UBI1* terminator was amplified using the primers ZJY019 and ZJY009 and then fused with the *UBI1* promoter fragment. Then, the linearized vector (pRPL40a-NAT or pUB-NAT) was integrated into the H99 *ubi1*Δ strain by biolistic transformation. Stable transformants were selected and confirmed by diagnostic PCR and quantitative real-time PCR. All the primers used in this study are listed in Supplementary Table S2.

### Southern Blot Analysis

Genomic DNA (20 μg) from each strain was extracted, using the CTAB method (Pitkin et al., 1996), and digested by the appropriate restriction endonucleases overnight. The DNA were separated on a 0.8% agarose gel, and then transferred to positively charged nylon membranes (Roche Applied Science, Indianapolis, IN, United States). The membranes were hybridized with the NEO or NAT probes overnight, washed, and then analyzed. The hybridized DNA bands were visualized on film after 3–10 min of exposure.

### RNA Preparation, Sequencing, and Quantitative Real-Time PCR

*Cryptococcus neoformans* was cultured in liquid YPD media until it reached mid-log phase. It was then pelleted, washed twice, frozen at −80°C, and lyophilized overnight. The cell pellets were broken with glass beads (2 mm diameter) and then the total RNA was isolated using the Qiagen RNeasy Plant Mini Kit (Qiagen, Valencia, CA, United States) following the manufacturer-provided protocol. Library preparation and RNA sequencing were performed by Genergy Biotech (Shanghai) Co., Ltd. The total RNA samples were purified and prepared as previously described (Trapnell et al., 2010). High-throughput sequencing was performed on a Hiseq3000 Genome Analyzer (Illumina, United States). To achieve sufficient sequence coverage, both the WT and mutant strains were sequenced with 100-bp paired-end reads. All the reads were mapped to the reference genome of *C. neoformans* from the Broad Institute website, using TopHat v1.3.0 (Trapnell et al., 2009). Less than 1% of the reads in all samples were excluded for poor quality. Among the aligned reads, the fragments per kilobase of transcript per million fragments mapped (FPKM) were counted to evaluate the transcriptional level, using Cufflinks v.1.0.3. The transcriptomic differences between the strains were analyzed and determined using CuffDiff (Trapnell et al., 2010). A gene was considered differently expressed if the transcriptional change was greater than 2.0-fold and the *P*-value less than 0.05 after Benjamini–Hochberg correction.

RNA was converted to cDNA with the SuperScript III First-Strand Synthesis Kit for RT-PCR (Invitrogen Corp., Carlsbad, CA, United States), according to the manufacturer’s instructions. Quantitative real-time PCR was performed to verify the RNA sequencing results using iQ SYBR Green Supermix (Bio-Rad, Hercules, CA, United States). The *ACT1* gene was utilized as an internal control to normalize gene amplification for each sample. The real-time PCR conditions were as follows: an initial denaturing cycle of 95°C for 3 min, followed by 40 cycles of denaturation at 95°C for 10 s, and annealing/extension at 60°C for 20 s. All primers used for the RT-PCR assays are listed in Supplementary Table S2.

### Growth Rate Assay, Colony Size Measurement, and Budding Rate Assay

To test the effect of *UBI1* deletion on growth rate, the WT, mutant (*ubi1*Δ), reconstituted (*ubi1*Δ*:UBI1*) and partially reconstituted (*ubi1*Δ*:RPL40a*) strains were grown overnight at 30°C in liquid YPD medium. The cell numbers were counted by hemocytometer and 10^6^ colony-forming units (CFUs) from each culture were transferred to flasks containing 30 mL fresh YPD medium and incubated at 30°C. Optical density at 600 nm (OD_600_) was measured for each culture at 4-h intervals, and growth curves plotted in Microsoft Excel. To detect the relationship between cryptococcal *UBI1* expression and its growth rate, 10^6^ CFUs of the WT and the P_CTR__4_*-UBI1* mutant strain were incubated under different conditions (YPD containing 25 μM CuSO_4_, YPD, and YPD containing 50 μM BCS) at 30°C. OD_600_ was recorded at each time point and growth curves plotted. Doubling time was used to evaluate the growth rate of the cryptococcal strains under distinct conditions, and calculated according to the formula: *T*_d_ = *t* × Lg2/(LgN_t_ − LgN_0_). *T*_d_, doubling time; *t*, culture time; *N*_t_, OD_600_ value after *t* hours of culture; *N*_0_, initial OD_600_ value at the time of inoculation.

For colony size measurement, different strains were grown in YPD liquid medium overnight at 30°C, washed twice with PBS buffer, and resuspended at a concentration of 10^3^ CFU per mL. -Each strain was spread (100 μL) on a YPD solid medium and grown at 30°C for 2 weeks. Photographs were taken on day 9 and day 14.

For the budding rate assay, 10^6^ CFU of each strain were inoculated into 30 mL YPD liquid medium and incubated to the mid-log (OD_600_ = 1.5) or stationary phase (OD_600_ = 4.0). Each culture was diluted to appropriate concentrations, and then stained with India ink. The budding yeast cells were then counted using an hemocytometer. Daughter cells were not considered budding cells if their size was larger than 50% of their parent cell size. The budding rate (%) was calculated with the formula: budding cells/total cells × 100. The total number of cells for each strain were not less than 500, and each test was repeated three times.

### Cell Cycle Analysis and Cell Size Measurement

For cell size measurement, 50 μL culture from each strain (WT, *ubi1*Δ, *ubi1*Δ*:UBI1*, and *ubi1*Δ*:RPL40a*) at mid-log phase was washed three times in 500 μL 1 × PBS, then resuspended in 100 μL 1 × PBS, and visualized with 25 μL India Ink. Images were collected using the 63× objective of a Zeiss LSM inverted confocal microscope (Jena, Germany), and the diameters of at least 100 cells were measured using Adobe Photoshop.

Cells (2 × 10^4^ cells for each strain) were harvested, fixed, and stained with propidium iodide (Sigma-Aldrich, St. Louis, MO, United States), and then analyzed using a BD FACSCalibur flow cytometer (Becton Dickinson Biosciences, Sparks, MD, United States). CellQuest Pro software was used for cell collection, and data analysis was performed using ModFit and FlowJo. The G1, S, and G2/M phases were identified using the Dean-Jett-Fox mathematical model.

### *In vitro* Phenotypic Assay

For capsule analysis, the WT, mutant, and reconstituted strains of *C. neoformans* were incubated in 1/10 Sabouraud medium at 37°C and 5% CO_2_ for 3 days (Zaragoza and Casadevall, 2004). Capsule measurements were performed as previously described (Maxson et al., 2007). Melanin production was visualized in cells spotted on L-DOPA agar and caffeic-acid medium at 30°C for 7 days (Fang et al., 2012). For titan-like cell induction, cryptococcal strains were incubated in Titan Cells Medium as previously described (Trevijano-Contador et al., 2018), which contains 5% Sabouraud and 5% fetal calf serum diluted in 50 mM MOPS (Ph7.3) plus 15 μM sodium azide (Sigma-Aldrich). For stress assays, each strain was cultivated to saturation in YPD medium, serially diluted (1–10^6^ dilutions), and spotted onto YPD or YNB agar media containing different stress-inducing agents, as previously reported (Fang et al., 2012).

### Transmission Electron Microscopy

The impact of *UBI1* deletion on the morphology of *C. neoformans* was evaluated with transmission electron microscopy (TEM). Cells of the WT, *ubi1*Δ, and *ubi1*Δ*:UBI1* strains were cultured to log phase in YPD liquid medium. The cells were harvested and fixed using the procedures developed by Reese et al. (2007). Ultrathin sections were cut using a Leica ultramicrotome, collected on copper grids, and stained with uranyl acetate and Satos lead. The samples were examined under a JEM-1230 transmission electron microscope (JOEL, Japan) using an acceleration voltage of 80 kV.

### Macrophage Killing Assay

The survival rate of the WT, *ubi1*Δ, *ubi1*Δ*:UBI1*, and *ubi1*Δ*:RPL40a* strains of *C. neoformans* within macrophage-like J744A.1 cells was assessed as previously described (Aslanyan et al., 2017). Macrophage cells (50 μL; 2 × 10^6^/mL) were aliquotted into the wells of a 96-well plate and activated by gamma interferon-c and lipopolysaccharide. The activated macrophages were co-incubated with 10^6^ CFU of a cryptococcal strain opsonized by a monoclonal antibody (C66441M, bought from Meridian Life Science, Inc.) for 2 h at 37°C in 5% CO_2_. The extracellular yeast cells were removed using PBS buffer and spread onto YPD agar for phagocytosis efficacy assessment. The monolayers were incubated in fresh DMEM media overnight, and then disrupted with 0.5% SDS to lyse the macrophages. The lysates were diluted and plated on YPD agar to calculate the viable fungal cells after incubation at 30°C for 3–5 days.

### *In vivo* Virulence Assessment, Fungal Burden Assay, and Histological Analysis

The animal protocol was approved by the Committee on Ethics of Biomedicine Research, Second Military Medical University. The protocol was carried out in strict accordance with the Guide for Care and Use of Laboratory Animals issued by the Chinese Ministry of Science and Technology. All efforts were made to minimize animal suffering and to reduce the number of mice used.

Female BALB/c mice (5 weeks old) were infected with the WT, *ubi1*Δ, or *ubi1*Δ*:UBI1* strains of *C. neoformans* by intranasal inoculation, according to an established protocol (Fang et al., 2012). For the survival assay, ten mice per group were infected with 10^5^ yeast cells suspended in 50 mL PBS. The infected animals were monitored and sacrificed based on predetermined endpoints, as previously described (Rittershaus et al., 2006). For the fungal burden assay, the lungs and brains were removed from the sacrificed mice (four mice for each group) after 3, 7, 14, and 21 days. Organ samples were weighed and homogenized. The homogenate (200 mL) was plated onto YPD agar with appropriate dilution, and incubated at 30°C for 3–5 days. The number of CFUs was calculated and expressed as CFU/g of tissue. For histological analysis, the lungs were removed from the infected mice at the indicated time points and fixed in 10% neutral buffered formalin. Paraffin sections (5 μm) of the lungs were stained with PAS (for *C. neoformans*) and counterstained with hematoxylin and eosin.

### Statistical Analysis

All values are expressed at mean ± S.D. unless otherwise indicated. Statistical analyses were performed using SPSS 18.0 (IBM, Armonk, NY, United States). Differences between paired groups were evaluated using analysis of variance and student’s *t*-test (two-tailed). Data from the survival assay in the murine infection model were analyzed using the Mantel–Cox test. *P-*values less than 0.05 were considered statistically significant.

## Results

### Identification of the Putative *C. neoformans UBI1* Gene

**FIGURE 1:**
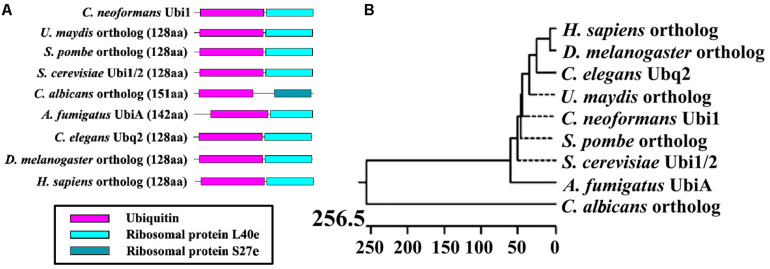
Bioinformatic analysis of putative Ubiquitin hybrid protein Ubi1. **(A)** Comparison of Ubi1 orthologs between *Cryptococcus neoformans* and other species. Ubi1 ortholog diagrams show functional protein domains, including a monoubiquitin and a ribosomal protein (L40e or S27e), which were identified by the Pfam database. **(B)** Phylogenetic analysis was depicted by Jotun Hein alignment using DNASTAR software. The protein sequences of putative Ubi1 orthologs were retrieved from the following databases: *C. neoformans* Ubi1 (CNAG_00370) from the *C. neoformans* H99 database, and its orthologs among other eukaryotic species from the protein databank of the NCBI website. XP_011388728, *Ustilago maydis* (Um); NP_593923, *Schizosaccharomyces pombe* (Sp); CAA86130 and CAA51949, *S. cerevisiae* (Sc); XP_715397, *Candida albicans* (Ca); XP_001481702, *Aspergillus fumigatus* (Af); NP_499695, *Caenorhabditis elegans* (Ce); NP_001260018, *Drosophila melanogaster* (Dm); NP_001307950, *Homo sapiens* (Hs). The bar marker indicates genetic distance.

A search of the *C. neoformans* genome database revealed that the *UBI1* (accession number CNAG_00370) coding region was 787 bp long with five exons and encodes a 129-amino-acid protein. The ubiquitin hybrid protein *UBI1* consisted of a monoubiquitin domain fused to a 53-aa C-terminal extension protein (Ribosomal unit protein L40a, Rpl40a) (Figure 1A). Other putative eukaryotic *UBI1* orthologs displayed an identical composition of domain structures, including the single ubiquitin moiety and the ribosomal protein L40e or S27e (Figure 1A). The putative sequence of Cn-Ubi1 was highly similar to its counterparts in other fungi (*Schizosaccharomyces pombe*, 96% identity; *S. cerevisiae*, 95% identity) and other eukaryotic species (*Caenorhabditis elegans*, 89% identity; *Drosophila melanogaster*, 88% identity) (Figure 1B), indicating that the Ubi1 protein is highly conserved across eukaryotic species.

### *UBI1* Deletion Severely Limits Growth in *C. neoformans*

**FIGURE 2:**
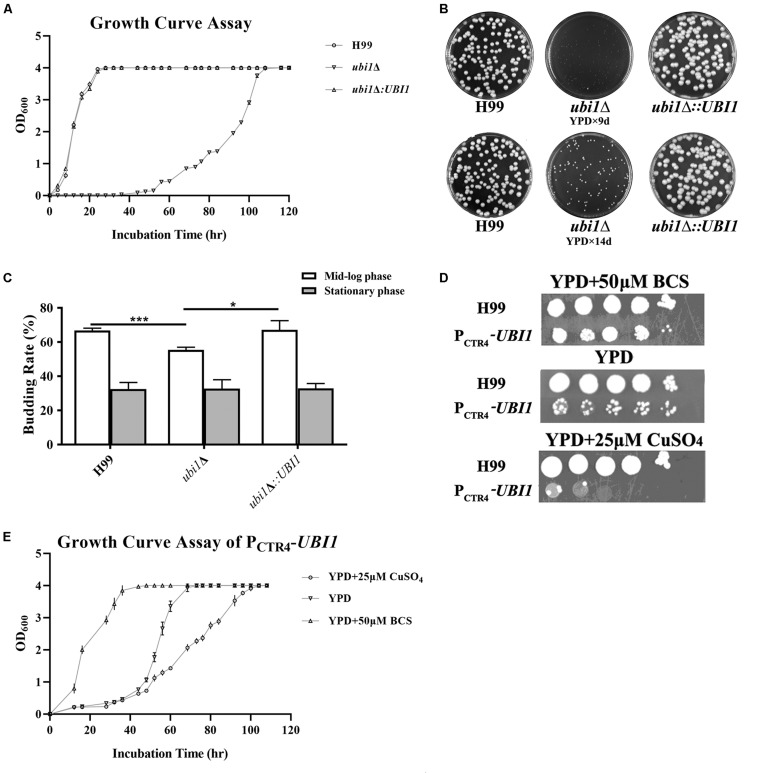
Ubi1 is essential for growth and proliferation in *C. neoformans*. **(A)** Growth rate assay. Samples (10^6^ CFU) of the wild-type (WT, H99), mutant (*ubi1*Δ), and reconstituted (*ubi1*Δ*:UBI1*) strains were transferred to 30 mL fresh YPD medium in flasks and incubated at 30°C. OD_600_ was measured for each culture at 4-h intervals. All assays were performed in triplicate. **(B)** Colony size measurement. Samples (100 CFU) of each strain were spread on YPD agar medium and then incubated at 30°C for 2 weeks. Photographs of each petri dish were taken on day 9 and day 14. **(C)** Budding rate assay. Each strain was incubated to the mid-log or stationary phase in YPD liquid medium at 30°C. Each culture was diluted and stained with India ink. The budding rates (%) were calculated with a hemocytometer using the formula: budding cells/total cells × 100 for each strain. The total number of cells for each strain was not less than 500, and each test was repeated three times. ***P < 0.001, *P < 0.05. **(D)** The growth performance of the condition-regulated strain (P*_*CTR*__4_*-*UBI1*) under YPD agar with/without CuSO_4_ or BCS. The WT and P*_*CTR*__4_*-*UBI1* mutants were grown to saturation in YPD liquid medium, 10-fold serially diluted, and 5 μL cells spotted on different YPD solid media. The samples were incubated at 30°C for 1 week and then photographed. **(E)** The growth rate of the condition-regulated strain (P*_*CTR*__4_*-*UBI1*) was inversely correlated with copper concentration and, thus, positively correlated with *UBI1* expression level.

To understand the function of the ubiquitin hybrid protein Ubi1 in *C. neoformans*, both deletion mutant (*ubi1*Δ) and reconstituted (*ubi1*Δ*:UBI1*) strains were constructed using a biolistic system and confirmed via diagnostic PCR and Southern Hybridization (Supplementary Figure S1). Intriguingly, the *ubi1*Δ mutant displayed severe growth restriction. Therefore, we first compared its growth rate with the WT (H99) and reconstituted strains in rich medium (YPD) at 30°C. After a short lag phase of 4 h, the H99 and *ubi1*Δ*:UBI1* strains rapidly entered the log phase, and they reached the stationary phase within 36 h. In contrast, the *ubi1*Δ mutant remained in the lag phase for 48 h and only reached the stationary phase after 108 h (Figure 2A). The generation times of the H99 and *ubi1*Δ mutant strains were 3.7 and 12.2 h, respectively, suggesting that Ubi1 is critical for maintaining growth rate in *C. neoformans*. In addition, the *ubi1*Δ mutant strain displayed significantly smaller colonies than either the H99 or *ubi1*Δ*:UBI1* strains after 9 or 14 days incubation on YPD agar at 30°C (Figure 2B). We next evaluated the budding rate of each strain (Figure 2C). At mid-log phase, the *ubi1*Δ mutant strain displayed about a 55.5% budding rate, significantly lower than those of the H99 (66.8%) or reconstituted (67.3%) strains (*P* < 0.001). At stationary phase, however, there were no statistical difference in the budding rate of each strain. Our data reveal that the ubiquitin hybrid protein Ubi1 is important for cryptococcal vegetative growth.

To further clarify the role of the ubiquitin hybrid protein Ubi1 inyeast growth, the copper-repressible promoter P*_*CTR*__4_* was inserted upstream of the *UBI1* gene to enable its expression in *C. neoformans* to be regulated by copper. Quantitative real-time PCR was performed to examine *UBI1* mRNA levels under the control of varying levels of copper to correlate with growth rate. Compared to H99 grown in regular YPD medium, the *UBI1* transcript showed a 66-fold reduction when grown in YPD containing CuSO_4_ but an 80-fold increase when grown in YPD containing BCS (Supplementary Figure S2A). At 30°C, H99 displayed normal growth in YPD medium and in YPD containing BCS or CuSO_4_, with a doubling time of about 4 h (3.7–4.2 h) in the different media (Supplementary Figure S2B). In contrast, the P*_*CTR*__4_* reconstituted strain (P_CTR__4_*-UBI1*) showed significant growth arrest in the YPD agar containing CuSO_4_, partial growth arrest in YPD, and normal growth in the YPD containing BCS, with doubling times of 11.6, 7.1, and 4.2 h for those three media, respectively (Figure 2E). The growth performance of P_CTR__4_*-UBI1* on YPD agar, with or without copper ions, was highly consistent with the results obtained from the liquid media (Figure 2D), further indicating that the cryptococcal proliferation rate is positively correlated with *UBI1* expression level.

### *UBI1* Deletion Impaires the Normal Morphology and Intracellular Structure of *C. neoformans*

**FIGURE 3:**
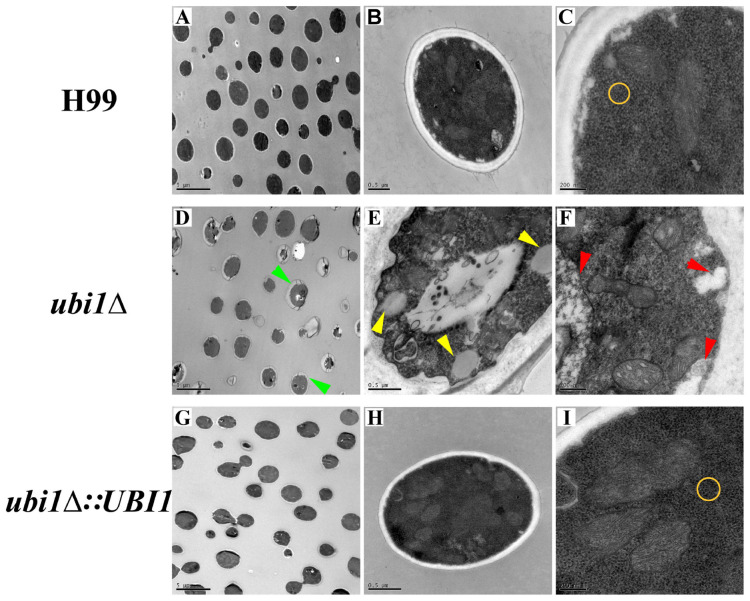
Impact of *UBI1* deletion on the cell morphology and intracellular structure of *C. neoformans.* The TEM images represent different strains as follows, H99 **(A–C)**, *ubi1*Δ **(D–F)**, and *ubi1*Δ*:UBI1* **(G,H)**. Sizes of the scale bar: 5 μm for **(A,D,G)**; 0.5 μm for **(B,E,H)**; and 200 nm for **(C,F,I)**. Green arrow, irregular cell shape and uneven cell wall thickness; red arrow, swelling mitochondria with dissoluted ridge; yellow arrow, intracellular vacuoles; yellow circle, comparison of ribosomal density.

Next, we studied the effects of Ubi1 on the cell morphology and intracellular structure of *C. neoformans* using TEM. Compared with the WT strain, deletion of *UBI1* caused several significant morphological alterations in *C. neoformans* cells, including irregularly shaped and larger cryptococcal cells (Figures 3A,D), uneven cell wall thickness, more projections or pseudopodia-like structures on the cell membrane (Figures 3D,E), fewer free ribosome particles (Figures 3C,F), more irregularly shaped mitochondria, and fewer cristae (Figure 3F). Moreover, *ubi1*Δ mutant cells had more sac-like or tubular cristae, whereas WT cells had more lamellar cristae (Figures 3C,F). When the *UBI1* gene was reconstituted, the cellular morphology and intracellular structure was completely restored to WT status (Figures 3G–I), indicating that the hybrid protein Ubi1 is essential for proper morphological maintenance and organelle functioning in *C. neoformans*.

### The Effect of Ubi1 on Cell Growth Is Mainly Conferred by Its C-terminal Ribosomal Protein Domain

**FIGURE 4:**
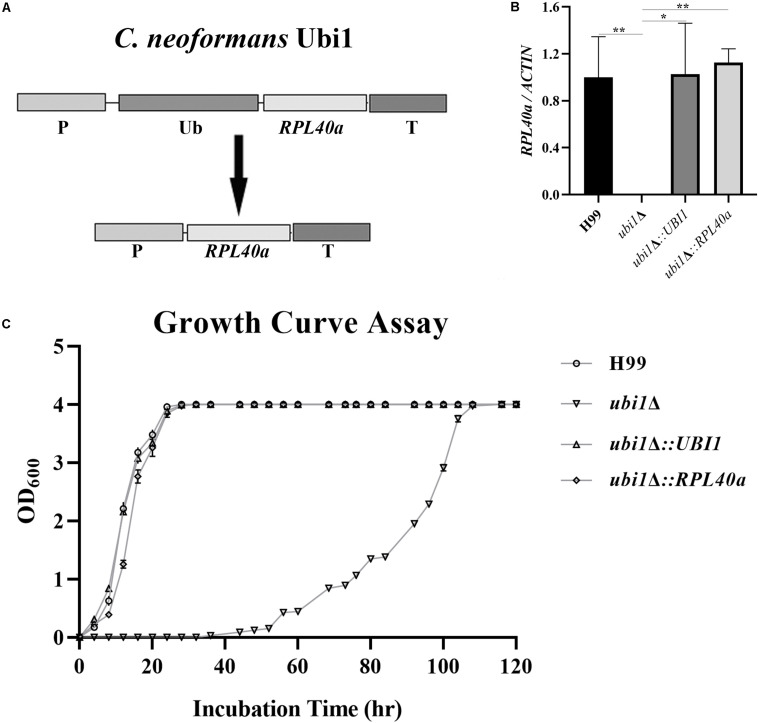
The C-terminal domain Rpl40e is responsible for the function of Ubi1 in regulating cryptococcal growth. **(A)** Construction of the partially reconstituted strain *ubi1*Δ*:RPL40a*. The truncated gene, *RPL40a*, containing the *UBI1* promoter, *RPL40a* ORF, and *UBI1* terminator, was cloned into the vector pCH233. The generated vector, pRPL40a-NAT, was linearized and integrated into the mutant strain *ubi1*Δ. **(B)** Confirmation of the partially reconstituted strain *ubi1*Δ*:RPL40a* via real-time PCR. The strain *ubi1*Δ*:RPL40a* displayed a similar transcriptional level to that of the WT (H99) or the fully reconstituted (*ubi1*Δ*:UBI1*) strain. The test was repeated three times for each strain. *P < 0.05, **P < 0.01. **(C)** After introduction of the *RPL40a* domain, the *ubi1*Δ mutant restored its growth rate, similar to that of WT or the fully reconstituted strain. Each experiment was performed in triplicate.

Ribosomes are key organelles that mediate protein translation, and thus determine the growth and proliferation of eukaryotic cells (Cheng et al., 2019). In *S. cerevisiae*, Rpl40e deletion induced a slow-growth phenotype, characterized by impaired 60S ribosomal subunit biogenesis (Finley et al., 1989; Fernández-Pevida et al., 2012). To investigate the roles of Rpl40e in regulating cryptococcal growth, we generated a truncated *UBI1*-targeting vector containing only the C-terminal *RPL40a* domain (Figure 4A) and transformed it into a *ubi1*Δ mutant strain. The reconstituted *ubi1*Δ*:RPL40a* strain was confirmed by diagnostic PCR and real-time PCR. The transcriptional levels of *RPL40a* were essentially the same between *ubi1*Δ*:RPL40a* and the WT or reconstituted (*ubi1*Δ*:UBI1*) strains (Figure 4B). Compared with the knockout strain *ubi1*Δ, the growth rate of the partially reconstituted strain *ubi1*Δ*:RPL40a* was significantly increased, and was similar to those of the WT and fully reconstituted *ubi1*Δ*:UBI1* strains (Figure 4C), indicating that the C-terminal *RPL40a* domain of Ubi1 is responsible for its function in regulating cryptococcal growth.

### Ubi1 Is Essential for the Cell Cycle Progression of *C. neoformans*

**FIGURE 5:**
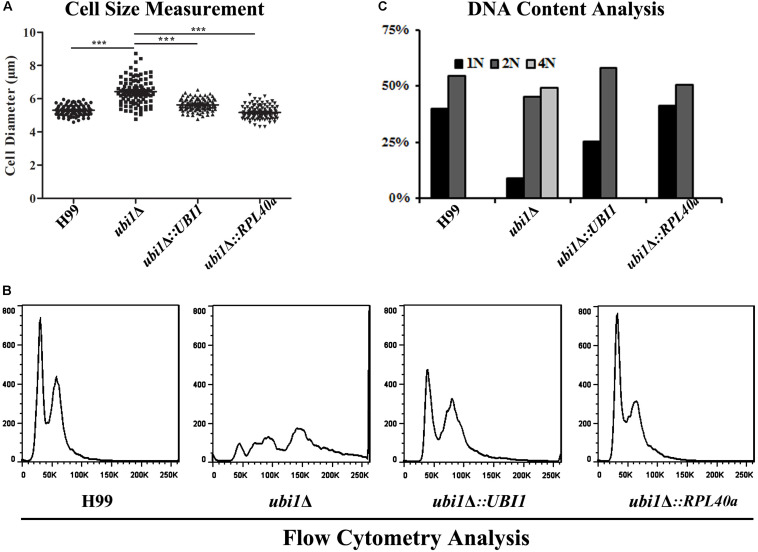
The C-terminal domain Rpl40e is responsible for the function of Ubi1 in regulating cryptococcal growth. **(A)** Deletion of Ubi1 led to enlargement of cell size in *C. neoformans*. Each strain was incubated to mid-log phase at 30°C, washed, and then visualized using the 63× objective of a Zeiss LSM inverted confocal microscope (Jena, Germany). ***P < 0.001. For each culture, the cell sizes of at least 100 cells were measured by using Adobe Photoshop Software. Flow cytometry analysis was performed to assess the effect of *UBI1* deletion on the ploidy **(B)** and cell cycle progression **(C)** of *C. neoformans*. Each experiment was performed in duplicate.

Cell size is a key factor for proliferative capacity and growth rate regulation in most eukaryotic cells. Cell size homeostasis is controlled at the G1/S phase boundary, primarily by preventing cell division until a “critical cell size” is attained (Aldea et al., 2017). The cell size of the *ubi1*Δ mutant strain of *C. neoformans* was therefore examined. When cultured to mid-log phase in rich medium (YPD) at 30°C, the average diameters of H99, *ubi1*Δ, *ubi1*Δ*:UBI1*, and *ubi1*Δ*:RPL40a* cells were 5.30 ± 0.30, 6.43 ± 0.76, 5.63 ± 0.36, and 5.17 ± 0.41 μm, respectively (*P* < 0.0001) (Figure 5A). Propidium iodide staining, followed by a fluorescence flow cytometry assay, showed that the deletion of *Ubi1* resulted in irregular DNA content in *C. neoformans*, which ranged from 1N to more than 4N (49%). Reconstitution of *UBI1* or *RPL40a* reversed the ploidy of the *ubi1*Δ cells back to a regular 1N/2N mode (Figure 5B). Moreover, normal cell cycle progression was blocked by *UBI1* deletion (manifested by the depletion of S phase cells), and was rescued by the introduction of either full-length Ubi1 or only *RPL40a* (Figure 5C).

### Ubi1 Is Involved in Capsule Production, Melanin Secretion, Titan-Like Cell Formation, and Stress Tolerance of *C. neoformans*

**FIGURE 6:**
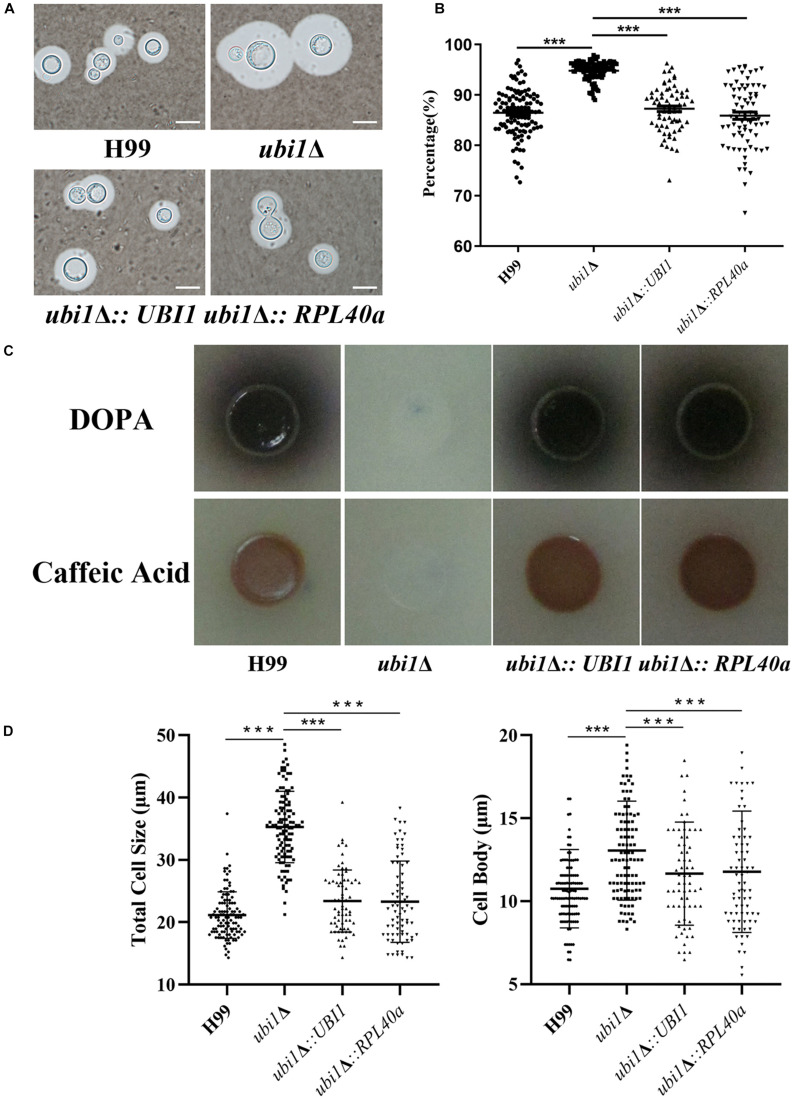
Ubi1 is involved in the production of capsule and melanin in *C. neoformans*. **(A)** Capsule induction assay. All the strains were cultured on DME medium at 37°C for 5 days. The capsules were examined by staining them with India ink and visualizing them at 100× magnification (scale bar = 5 μm). **(B)** Relative capsule volume detection. Total (cell and capsule) and cell-only diameter were measured via Adobe Photoshop Software for more than 50 cells of each strain. Then, the relative ratio of the capsule was calculated with the formula: (Total Volume-Packed Volume)/Total Volume. **(C)** Melanin production assay. Strains were grown on L-DOPA or caffeic-acid medium for 5 days at 30°C. **(D)** Titan-like cell induction assay. Strains were grown on Titan Cells Medium at 37°C and 5% CO_2_ for 3 days. Measurements of total cell sizes (left) and cell body sizes (right). ***P < 0.001.

Since capsules and melanin are important pathogenic factors for cryptococcal invasion into the host, we further examined their expression in each strain. As shown in Figure 6, *UBI1* deletion induced a slight increase in the capsule production of *C. neoformans* (Figures 6A,B, *P* < 0.001), but fully blocked its melanin secretion. Reconstitution of the *UBI1* or *RPL40a* gene restored the normal production of both capsules and melanin in the *ubi1*Δ mutant. Besides, we also evaluated the effect of *UBI1* deletion on the formation of titan cells, which confers distinct advantages to *C. neoformans* against the host during infection (Zaragoza and Nielsen, 2013). After incubation in Titan Cells Medium, the *ubi1*Δ mutant exhibited a significant enlargement of total cell size and cell body size compared to WT or reconstituted strain (Figure 6D; *P* < 0.001), indicating that Ubi1 is involved in titan-like cell formation *in vitro*.

To examine the roles of Ubi1 in stress response, different *C. neoformans* strains were exposed to various stresses. High temperature, oxidative stress (H_2_O_2_ and NaNO_2_), sorbitol, high salt (NaCl and KCl), Congo red, and SDS all exacerbated the growth restriction or growth defect of the *ubi1*Δ strain (Supplementary Figure S3). Both the *ubi1*Δ*:UBI1* and *ubi1*Δ*:RPL40a* strains exhibited growth similar to the WT (H99) in the presence of the above-mentioned stressors (Supplementary Figure S3). Together, these phenotypic assays *in vitro* suggest that *Ubi1* may be essential for cryptococcal adaptability to various environmental niches.

### Ubi1 Is Required for Virulence in *C. neoformans*

**FIGURE 7:**
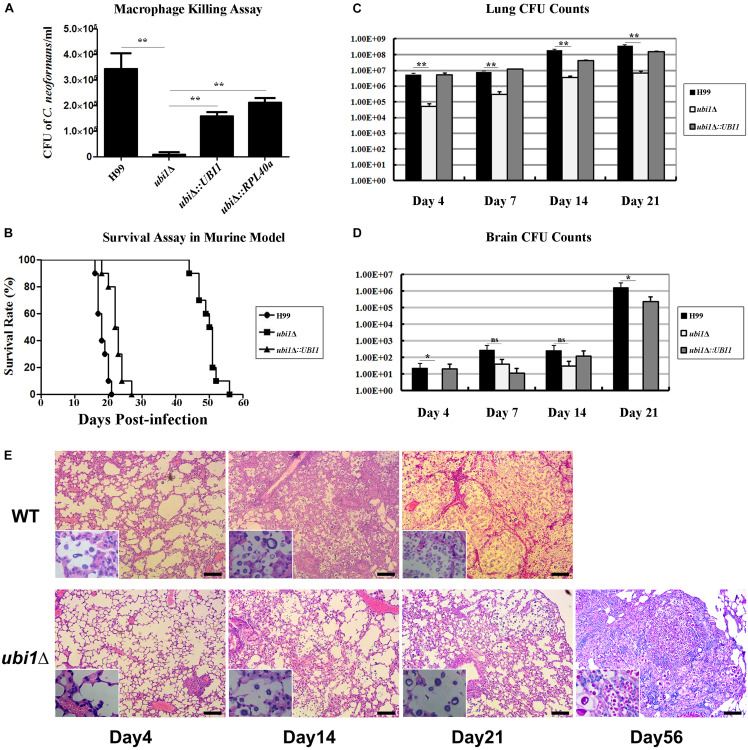
Ubi1 is required for *C. neoformans* virulence. **(A)** *C. neoformans* colonies were counted after uptake and killing by activated macrophages overnight and macrophage lysate was diluted and cultured on YPD plates. **P < 0.01. **(B)** The mouse survival assay showed that *ubi1*Δ *C. neoformans-*infected mice had significantly longer survival times (*P* < 0.01). Mouse lung **(C)** and brain **(D)** fungal burdens were evaluated by counting fungal colonies after culturing serially diluted tissue lysate on YPD plates. *P < 0.05, **P < 0.01; ns, no significant difference. **(E)** Ubi1 deletion altered host pulmonary inflammation responses to *C. neoformans.* Representative photographs of hematoxylin and eosin-stained mouse lungs at different times post-infection. Scale bar = 100 μm.

Next, we examined the effect of *UBI1* deletion on cryptococcal virulence via macrophage killing and mouse survival assays. WT H99, *ubi1*Δ, and reconstituted strains (*ubi1*Δ*:UBI1* and *ubi1*Δ*:RPL40a*) were co-cultured with IFN-γ and LPS-activated J774A.1 macrophages. There were no differences among the strains in the initial uptake into the macrophages (data not shown). However, deletion of the *Ubi1* gene reduced the intracellular survival of *C. neoformans* by about 97% (*P* < 0.0001 vs. H99), while reconstituting *UBI1* or *RPL40a* partially restored its intracellular survival rate (Figure 7A).

The *in vivo* virulence of the WT, *ubi1*Δ, and *ubi1*Δ*:UBI1* strains was evaluated using a mouse survival assay. Mice infected with the *ubi1*Δ strain exhibited prolonged survival (49.8 ± 3.3 days, *P* < 0.001 compared to WT) while the average survival times of WT- and *ubi1*Δ*:UBI1*-infected mice were 18.3 ± 1.6 and 22.5 ± 2.4 days, respectively (Figure 7B), indicating a significant role of Ubi1 in the pathogenesis of *C. neoformans*. Since pneumonia and meningoencephalitis were the primary clinical manifestations of cryptococcosis, the fungal burdens of the lungs and brain of *C. neoformans*-infected mice were assessed. As shown in Figure 7C, the cryptococcal burden gradually increased in the lungs of mice infected by the *ubi1*Δ mutant, but was significantly lower (about 30–110 fold) than those of WT- and *ubi1*Δ*:UBI1*-infected mice (Figure 7C). The brain fungal burden of WT- and *ubi1*Δ*:UBI1*-infected mice remained low until 21 days post-infection (pi). However, *ubi1*Δ infection produced a low brain fungal burden 7 and 14 days pi and almost no detectable living yeast cells 4 days or 21 days pi (Figure 7D). These results indicate that *UBI1* deletion might lead to a chronic pulmonary infection, with delayed or reduced brain invasion by *C. neoformans*.

### *UBI1* Deletion Alters Mouse Pulmonary Inflammation and Immune Responses Toward *C. neoformans*

As the mice infected by the *ubi1*Δ strain displayed significantly prolonged survival, but finally succumbed to chronic pulmonary infection, we next assessed the effects of *UBI1* deletion on the pathological changes in mouse lung and immune responses to *C. neoformans*. In BALB/c mice infected with the WT strain, a few yeast cells were confined to the alveolar space or septum, along with mild infiltration of the neutrophils, at the early stage of infection (day 4 pi; Figure 7E). On day 14 pi, mild destruction of the alveolar architecture owing to propagated cryptococci and severe inflammation were observed. The inflammatory cells were primarily mononuclear cells with a few eosinophils. Widespread pulmonary alveolar destruction and a large quantity of fungi with much fewer inflammatory cells were seen 21 days pi-.

Mouse lungs infected with the *ubi1*Δ mutant strain exhibited a different histological pattern. On day 4 pi, limited yeast cells were present in the alveolar septum, accompanied by increased inflammatory cell infiltration. Intriguingly, on days 14 and 21 pi, there was a significant increase of fungal cells present in the alveolar space but without any exacerbation of inflammation. By day 56 pi, however, a large proportion of the pulmonary alveoli were destroyed and filled with a multitude of yeast cells in the presence of mononuclear and lymphoid cells. It was also noted that a proportion of titan cells, an important *C. neoformans* morphotype characterized by enlarged cells with extensive capsules, were observed at the late stage of infection (day 56 pi) which occurred around 14 days pi in the lungs of mice infected with the WT strain (Figure 7E).

**FIGURE 8:**
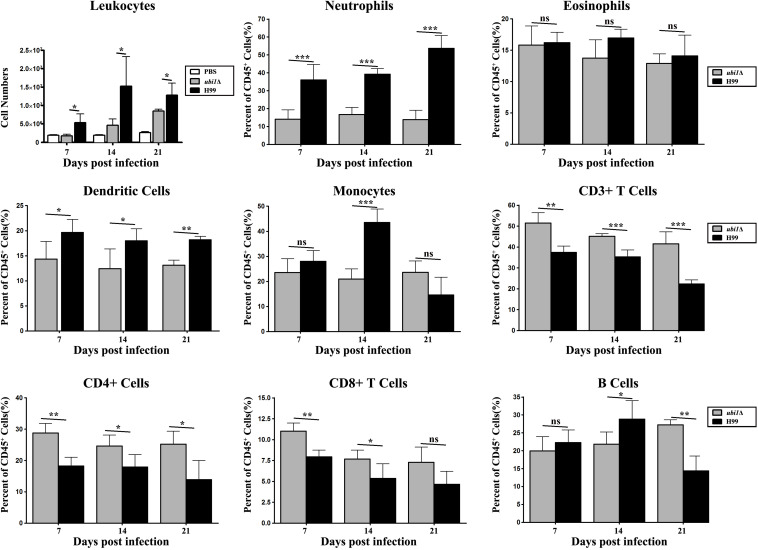
Ubi1 regulated pulmonary recruitment of leukocyte subsets during cryptococcal infection. Different leukocytes and lymphocytes from mice infected by *C. neoformans* were assessed by flow cytometry. Results represent mean ± SEM, *N* = 4 or more mice per group. *P < 0.05, **P < 0.01, ***P < 0.001; ns, no significant difference.

**FIGURE 9:**
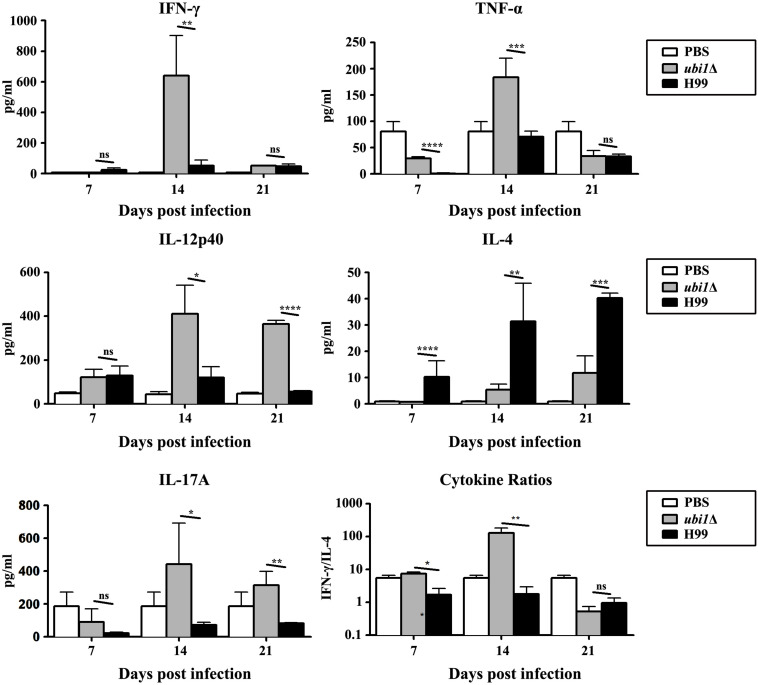
*UBI1* deletion induced the robust development of pulmonary Th1 cytokine bias during cryptococcal infection. Specified inflammatory cytokines of *C. neoformans-*infected mice were analyzed using ELISA. Results represent mean ± SEM, *N* = 4 or more mice per group. *P < 0.05, **P < 0.01, ***P < 0.001, ****P < 0.0001; ns, no significant difference.

In order to characterize the effect of cryptococcal *UBI1* deletion on pulmonary inflammatory response, we assessed changes in the immune cell population using flow cytometry with total lung cells on days 7, 14, and 21 pi. Mouse lungs infected with the *ubi1*Δ strain had significantly fewer total leukocytes, neutrophils, and dendritic cells than the WT strain-infected mouse lungs at all time points. Monocyte numbers were substantially higher in the WT-infected mouse lungs only on 14 days pi, while the number of eosinophils was not different at any time point. The total numbers of T cells, CD4^+^ T cells, and CD8^+^ T cells were markedly higher in the *ubi1*Δ strain-infected mouse lungs than in the WT-infected ones at all infection stages. The B cells showed a biphasic change pattern; the *ubi1*Δ strain-infected mouse lungs had fewer B cells 14 days pi but significantly more B cells 21 days pi than the WT-infected mouse lungs (Figure 8). Accordingly, mice produced different patterns of cytokines upon infection with WT and *ubi1*Δ *C. neoformans*. The levels of TNF-α, IL-20p40, and IL-17A in the *ubi1*Δ strain-infected mice were much higher than those in the WT-infected mice at all examined time points. However, the IL-4 level was drastically higher in the WT-infected mice at every stage of infection. The IFN-γ level was higher in the WT-infected mice 7 days pi but in the *ubi1*Δ *C. neoformans-*infected mice 14 days pi (Figure 9).

### *UBI1* Deletion Alters the Transcriptome of *C. neoformans*

To explore the molecular basis for the defect of *in vitro* growth and *in vivo* invasion caused by *UBI1* deletion, RNA sequencing was performed to compare the transcriptional profiles of WT and *ubi1*Δ cells. The data have been submitted to the GEO database under accession number GSE142374^2^. A total of 236 genes were differentially expressed (141 upregulated and 95 downregulated) by at least twofold (*P* < 0.05) between these two strains (Table 1). The genes regulated by *UBI1* deletion covered a variety of functional categories. First, a group of genes involved in ribosome biogenesis and RNA processing and transport were significantly upregulated in the *ubi1*Δ mutant compared to the WT. Consistent with the reduced ribosome particles in the TEM assay (Figure 3), these results suggest that *UBI1* deletion might induce ribosome stress and dysfunction in *C. neoformans*. Furthermore, several genes involved in nutritional metabolism were differentially expressed by the *UBI1* mutation. Several enzymes in amino acid metabolism (except the sulfur-containing amino acids), steroid biosynthesis, and oxidative phosphorylation were repressed, suggesting that *UBI1* deletion might alter metabolic flux in *C. neoformans*. Ubi1 inactivation also resulted in the downregulation of genes associated with cell cycle progression (such as *TEM1* and *CDC31*) and nucleotide metabolism, which might partially explain the cell cycle arrest in the *ubi1*Δ mutant (Figure 5). Finally, *UBI1* deletion also led to the differential expression of several genes involved in other basic cellular processes, such as DNA repair, protein processing, and signaling pathways.

**TABLE 1:** Ubi1-dependent gene expressions in *Cryptococcusneoformans*.

**GO category**	**Gene ID**	**Folds**	***P*-value**	**Functional annotation**
Ribosome biogenesis	CNAG 01608	11.79	0.0074	Small GTP-binding protein domain
	CNAG 06468	11.42	0.0061	Nucleolar GTP-binding protein 1
	CNAG 06919	9.78	0.0032	rRNA 2′-*O*-methyltransferase fibrillarin
	CNAG 06766	8.27	0.0367	Utp14 protein
	CNAG 05976	6.83	0.0426	Nucleolar protein 58
	CNAG 00449	5.74	0.0157	Nin one binding Zn-ribbon like
	CNAG 03602	5.64	0.0161	NUC189 domain-containing protein
	CNAG 01715	5.20	0.0240	AARP2CN domain-containing protein
	CNAG 06005	5.10	0.0195	Hypothetical protein
	CNAG 06472	4.96	0.0240	RNA recognition domain-containing protein
	CNAG_06273	4.84	0.0266	Ribosome biogenesis regulatory protein
	CNAG 00775	4.63	0.0452	U3 small nucleolar RNA C terminal
	CNAG 06811	0.13	0.0115	Ribosomal L22e protein family
RNA processing and transport	CNAG_00809	14.232824	0.00205	ATP-dependent RNA helicase ded1
	CNAG 07676	10.34	0.0017	ATP-dependent RNA helicase DBP2-A
	CNAG_00166	7.3819911	0.009	ATP-dependent RNA helicase DBP8
	CNAG_00603	7.1017275	0.0066	Pre-rRNA-processing protein PNO1
	CNAG 00785	6.41	0.0148	ATP-dependent RNA helicase eIF4A
	CNAG_04032	4.3295287	0.03825	ATP-dependent metallopeptidase HflB
	CNAG 06585	4.30	0.0307	Sec63 Brl domain-containing protein
	CNAG_03205	3.8616569	0.0471	ATP-dependent RNA helicase ROK1
	CNAG 06779	0.24	0.0463	Small nuclear ribonucleoprotein Sm D1
	CNAG 01666	0.18	0.0291	U6 snRNA-associated Sm-like protein LSm2
DNA repair	CNAG 01163	5.99	0.0390	SNF2 family domain-containing protein
	CNAG 03654	4.02	0.0496	RecQ family ATP-dependent DNA helicase
	CNAG 00991	0.21	0.0254	XPG domain-containing protein
Nucleotide metabolism	CNAG 00441	4.29	0.0400	Inosine-5′-monophosphate dehydrogenase IMD3
	CNAG 02847	0.19	0.0408	Thymidylate kinase
	CNAG 01395	0.16	0.0467	Ribose-5-phosphate isomerase
	CNAG 04307	0.13	0.0165	uricase
	CNAG 01915	0.12	0.0045	Ribonucleoside-diphosphate reductase small subunit
	CNAG 04692	0.12	0.0196	Thymidylate synthase
	CNAG 06694	0.05	0.0011	Hydroxyisourate hydrolase
Amino acid metabolism	CNAG 04136	10.79	0.0097	Methylthioribose-1-phosphate isomerase Mri1
	CNAG 00021	4.93	0.0477	Adenosylmethionine decarboxylase Spe2
	CNAG 00886	4.57	0.0311	Adenosylhomocysteinase Sah1
	CNAG 07908	4.54	0.0341	Aconitate hydratase 2
	CNAG 05611	0.21	0.0264	Pyridoxal-phosphate dependent enzyme
	CNAG 05584	0.20	0.0244	Methylthioribulose-1-phosphate dehydratase
	CNAG 06448	0.11	0.0111	Cys/Met metabolism PLP-dependent enzyme
	CNAG 03134	0.10	0.0097	3-hydroxyacyl-CoA dehydrogenase Fox2
Protein processing	CNAG 03944	9.46	0.0109	DnaJ central domain-containing protein
	CNAG 06150	6.45	0.0493	Heat shock protein Hsp90
	CNAG 04851	6.24	0.0231	Transitional endoplasmic reticulum ATPase
	CNAG 05846	5.83	0.0130	Ubiquitin-conjugating enzyme
	CNAG 01151	0.17	0.0302	Ubiquitin-conjugating enzyme
	CNAG 01279	0.13	0.0166	Ubiquitin-conjugating enzyme
Steroid biosynthesis	CNAG 01129	0.21	0.0239	Squalene/oxidosqualene cyclase
	CNAG 02830	0.15	0.0082	Ergosterol biosynthesis ERG4/ERG24 family protein
	CNAG 06829	0.12	0.0042	Squalene epoxidase
Oxidative phosphorylation	CNAG 02961	0.23	0.0343	ATP synthase subunit J
	CNAG 03025	0.10	0.0023	V-type proton ATPase subunit D
Endocytosis	CNAG 04311	5.67	0.0357	Snf7
	CNAG 04002	0.24	0.0438	BAR domain-containing protein
Peroxisome	CNAG 06551	5.06	0.0210	Choline/Carnitine *O*-acyltransferase
Cell cycle	CNAG 05513	0.09	0.0067	Septum-promoting GTP-binding protein Tem1
	CNAG_05655	0.08	0.00285	Cell division control protein Cdc31
Signaling pathway	CNAG 05890	0.16	0.0232	Guanine nucleotide-binding protein subunit gamma
Others	CNAG 01442	5.63	0.0270	Cysteine desulfurase
	CNAG 01539	0.14	0.0174	Myo-inositol-1-phosphate synthase
	CNAG 06659	0.11	0.0051	Glycosyl hydrolase family 20

## Discussion

Ubiquitin is a highly conserved post-translational modifier, involved in a variety of cellular processes (Clague and Urbé, 2010). In most eukaryotes, ubiquitin is expressed as two classes of proteins: (i) a hybrid protein between a ubiquitin monomer and a ribosomal protein (such as Rpl40e or Rps31e), and (ii) a polyubiquitin precursor including a polymer of several tandem ubiquitin monomers. The current study characterized the roles of the ubiquitin hybrid protein, Ubi1, in the growth and virulence of *C. neoformans*. Deletion of *UBI1* resulted in growth inhibition, cell ploidy increase, morphological abnormalities, cell cycle arrest, and attenuated virulence, which were all corrected by reconstituting *ubi1*Δ cells with either the full-length *UBI1* gene or the C-terminal *RPL40a* domain.

The most striking phenotypic observation in the cryptococcal *ubi1*Δ strain was the dramatic growth restriction, even under rich growth conditions. Several lines of evidence strongly support that the growth rate defect caused by *UBI1* deletion is closely associated with abnormal ribosomal biosynthesis. First, the TEM assay showed a significant reduction of ribosomes with sparse distribution in the *ubi1*Δ cells, compared with the WT or *ubi1*Δ*:UBI1* strains of *C. neoformans*. A deficiency of ribosomes, the conserved molecular machines that mediate protein translation, is closely associated with decreased bulk protein synthesis and thus a defective cell growth rate (Steffen et al., 2012; Cheng et al., 2019). Furthermore, reconstitution of the *UBI1* or *RPL40a* domain restored the exponential growth rate in cryptococcal strains with *UBI1* deletion. Rpl40e is a highly conserved large subunit ribosomal protein in eukaryotes (Finley et al., 1989; Kobayashi et al., 2016). In *S. cerevisiae*, Rpl40e is generated by the proteolytic cleavage of ubiquitin precursor proteins (Ubi1 and Ubi2) and is required for 60S subunit production, subunit assembly, and the translocation process during translation elongation (Finley et al., 1989; Fernández-Pevida et al., 2012). Deletion of either *RPL40*-encoding gene was characterized by a slow-growth phenotype, while double deletions caused a lethal phenotype in *S. cerevisiae* (Finley et al., 1989). Our transcriptome analysis further revealed that *UBI1* deletion in *C. neoformans* induced the significant upregulation of several genes involved in ribosome biogenesis and RNA processing and transport. Therefore, we speculate that Ubi1 might play similar roles in the ribosomal biogenesis and homeostasis of *C. neoformans*. The detailed mechanism requires further investigation.

In addition to growth rate, we found that Ubi1 also participated in regulating cell ploidy variation, morphological homeostasis, and cell cycle progression in *C. neoformans*. *UBI1* deletion induced a substantial increase of cryptococcal cells with 4N DNA content. Ploidy changes usually influence basic cellular properties, including cellular morphology. For example, cryptococcal Titan cells (highly polyploid cells) have a gigantic cell size (>10 μm), an enlarged capsule, and a dramatically thicker cell wall (Zaragoza et al., 2010; Dambuza et al., 2018). Consistent with the ploidy increase, the *ubi1*Δ strain also exhibited significant morphological alterations, such as enlargement of the cell size and extracellular capsule, uneven cell wall thickness, and more pseudopodia-like structures on the cell membrane. The ploidy alterations might be associated with the cell cycle arrest observed in *ubi1*Δ cells. Previous studies revealed that abrogation of ribosome biogenesis generated cell cycle arrest (Gómez-Herreros et al., 2013; Thapa et al., 2013; Polymenis and Aramayo, 2015). In *S. cerevisiae*, the repression of nine 60S ribosomal protein genes, including *RPL40a*, leads to cell cycle arrest in the G2/M phase (Thapa et al., 2013). Similarly, we observed that cell clusters with >2N DNA content accounted for 49% of *ubi1*Δ cells, suggestive of a G2/M cell cycle phenotype in *C. neoformans*. The phenotype is consistent with the transcriptomic profile of the *ubi1*Δ strain, in which expression of some proteins required for the G2/M phase of progression, such as Tem1 and Cdc31, were significantly downregulated (Shirayama et al., 1994; Ivanovska and Rose, 2001). Furthermore, the depletion of S phase cells was another cell cycle phenotype of the *ubi1*Δ strain. It also exhibited remarkable inhibition of the transcription of several genes involved in nucleotide anabolism of *C. neoformans*. Together, these data highlight the importance of Ubi1 (especially Rpl40e) in regulating cell ploidy regularity, morphological homeostasis, and cell cycle progression.

Finally, *UBI1* deletion induced a dramatic attenuation in the survival and infectivity of *C. neoformans* in mammalian hosts. Our macrophage and mouse experiments exhibited significantly reduced intracellular survival and fungal burdens for the *ubi1*Δ strain, which is consistent with the results of the stress assay. The phenotype is probably attributable to the growth rate defect of cryptococcal mutant cells owing to ribosomal deficiency. Intriguingly, growth *in vivo* of the *ubi1*Δ strain seemingly outpaces the WT strain in the early stage of pulmonary infection (Figure 7C). The difference of growth rates *in vivo* and *in vitro* is confounding, which requires further exploration. In addition, the attenuated virulence of the *ubi1*Δ strain was closely associated with the induction of a T-helper (Th)1-type response, which could promote the elimination of intracellular pathogens (Arora et al., 2011; Davis et al., 2013). Histology sections demonstrated more localized and mild inflammation in the lungs of *ubi1*Δ-infected animals, and presented infiltration with fewer total leukocytes, neutrophils, and dendritic cells but a higher proportion of T lymphocytes than mice infected with the WT strain. Furthermore, *ubi1*Δ-infected mice showed a significant increase of Th1-type cytokines (such as IFN-γ, TNF-α, IL-12, and IL-17A) while mice in the WT group showed an accumulation of the anti-inflammatory cytokine IL-4. This distinct immune response profile might be attributed to cryptococcal morphological alterations owing to *UBI1* deletion. The irregular shapes and uneven cell walls might significantly alter pathogen-associated molecular pattern exposure in *ubi1*Δ cells. For example, the *ubi1*Δ mutants showed a drastic defect in melanin secretion, and cryptococcal laccase (melanin synthase) induces immune modulation from Th1/Th17 responses toward Th2 responses (Qiu et al., 2012). Despite the attenuated virulence, however, the *ubi1*Δ strain eventually caused delayed lethality in mice, which probably died of chronic pulmonary infection. Previous reports revealed that the deletion of genes encoding 60S subunit proteins, including Rpl40e, leads to lifespan extension in *S. cerevisiae* (Steffen et al., 2008, 2012). *UBI1* deletion might also enhance longevity in *C. neoformans*, which would ultimately alter its survival and pathogenic patterns in the mammalian host.

In eukaryotes, ubiquitin is usually expressed as a hybrid protein with a fused ribosomal protein, such as Rpl40e or Rps31e (Clague and Urbé, 2010). The ubiquitin moiety could act as a molecular chaperone to the ribosomal proteins by facilitating their efficient production, folding, and ribosome assembly (Lacombe et al., 2009; Martín-Villanueva et al., 2019, 2020). Expression of *ubi1*Δ*ub*-HA, as the only source of Rpl40e, caused a severe slow-growth phenotype and aggregation of ribosomal proteins in *S. cerevisiae* (Martín-Villanueva et al., 2019). In the current study, however, partial reconstitution of the Rpl40e domain could rescue various abnormal phenotypes of the cryptococcal *ubi1*Δ strain, such as growth rate defect, cell ploidy variation, cell cycle arrest, and attenuated intracellular survival. Our work reveals the evolutionary convergence and divergence of the ubiquitin hybrid protein in different fungal species. The role of the ubiquitin moiety in the ribosome biogenesis, growth, and pathogenicity of *C. neoformans* requires further investigation.

## Conclusion

In summary, we demonstrated that the ubiquitin hybrid protein is required for maintaining vegetative growth, morphological homeostasis, cell cycle progression, and pathogenicity *in vivo* of *C. neoformans*. The Rpl40e component could abrogate the effects of *UBI1* deletion on cryptococcal growth and virulence, strongly supporting the importance of the ubiquitin hybrid protein in the ribosome biogenesis of *C. neoformans*.

## Data Availability Statement

The datasets generated in this study can be found in online repositories. The names of the repository/repositories and accession number(s) can be found in the article/Supplementary Material.

## Ethics Statement

The animal study was reviewed and approved by Committee on Ethics of Biomedicine Research, Second Military Medical University.

## Author Contributions

JZ, YY, YF, JG, and WF conceived, designed, and performed the experiments. JY, CZ, ZG, and WF analyzed the data. JG, WL, and WF drafted the manuscript. All authors approved the final version of the manuscript.

## Conflict of Interest

The authors declare that the research was conducted in the absence of any commercial or financial relationships that could be construed as a potential conflict of interest.

## Footnotes

1
https://www.ncbi.nlm.nih.gov/Taxonomy/Browser/wwwtax.cgi?id=235443

2
https://www.ncbi.nlm.nih.gov/gds/
