# Corrigendum: Ribosomal protein L40e fused with a ubiquitin moiety is essential for the vegetative growth, morphological homeostasis, cell cycle progression, and pathogenicity of *Cryptococcus neoformans*

**DOI:** 10.3389/fmicb.2024.1545744

**Published:** 2025-01-09

**Authors:** Jingyu Zhao, Yali Yang, Yibin Fan, Jiu Yi, Chao Zhang, Zhongkai Gu, Weihua Pan, Julin Gu, Wanqing Liao, Wei Fang

**Affiliations:** ^1^Shanghai Key Laboratory of Molecular Medical Mycology, Department of Dermatology, Changzheng Hospital, Second Military Medical University, Shanghai, China; ^2^Department of Dermatology, Shanghai Eastern Hepatobiliary Surgery Hospital, Shanghai, China; ^3^Department of Dermatology, Shanghai Ninth People's Hospital, School of Medicine, Shanghai Jiao Tong University, Shanghai, China; ^4^Department of Dermatology, Zhejiang Provincial People's Hospital, People's Hospital of Hangzhou Medical College, Hangzhou, China; ^5^The Institute of Biomedical Sciences, Fudan University, Shanghai, China

**Keywords:** *Cryptococcus neoformans*, ubiquitin, growth restriction, cellular morphology, virulence, immune evasion

In the published article, there was an error in Figure 3B as published. Upon recent review of our experimental data, we discovered that Figure 3B in this manuscript incorrectly used the same data as Figure 3B in our previous paper, “Pd@Ag Nanosheets in Combination with Amphotericin B Exert a Potent Anti-Cryptococcal Fungicidal Effect” (PMID: 27271376). The corrected Figure 3 and its caption appear below.

**Figure 3:**
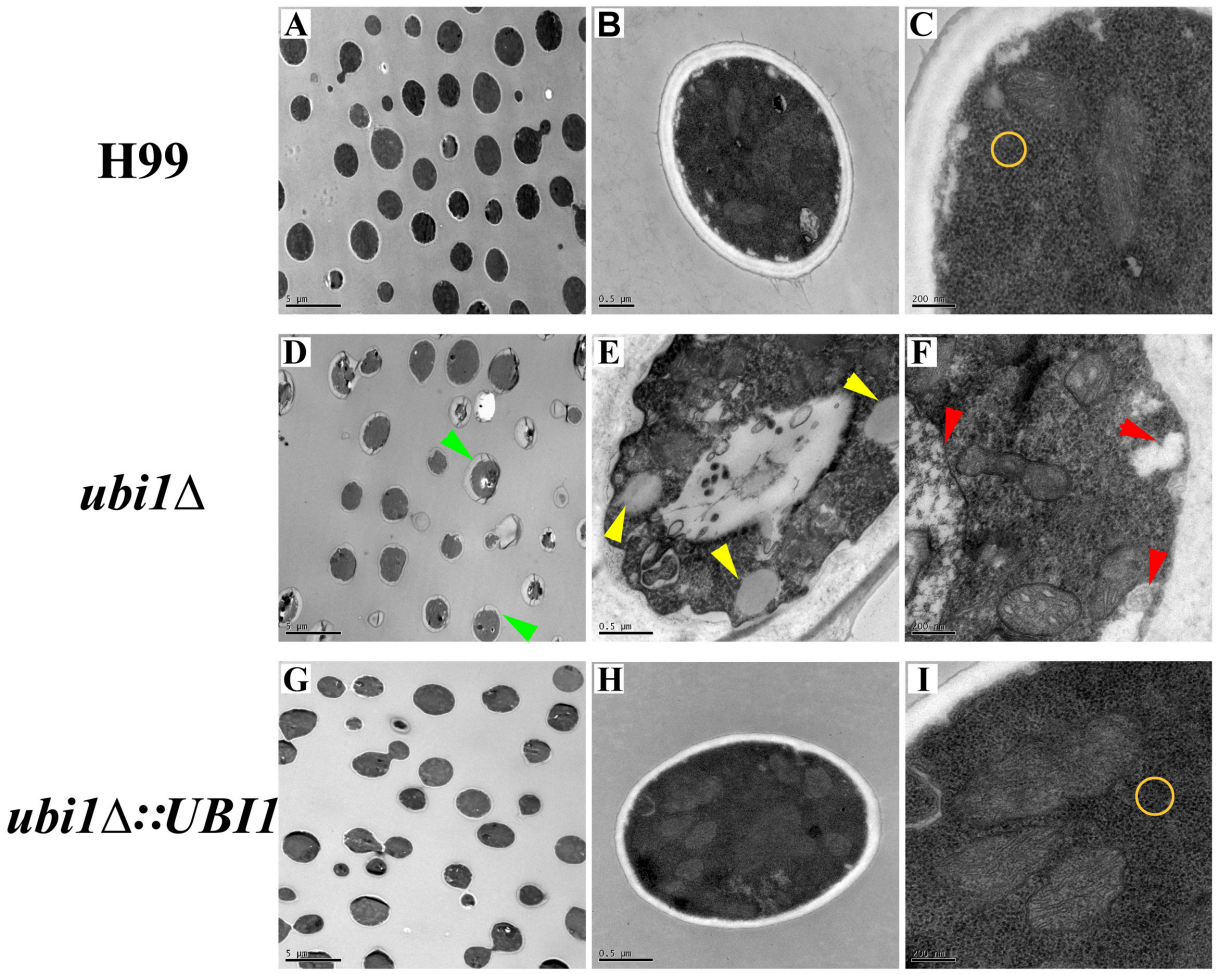
Impact of UBI1 deletion on the cell morphology and intracellular structure of *C. neoformans*. The TEM images represent different strains as follows, H99 **(A–C)**, *ubi1*Δ **(D–F)**, and *ubi1*Δ*:UBI1* **(G, H)**. Sizes of the scale bar: 5 μm for **(A, D, G)**; 0.5 μm for **(B, E, H)**; and 200 nm for **(C, F, I)**. Green arrow, irregular cell shape and uneven cell wall thickness; red arrow, swelling mitochondria with dissoluted ridge; yellow arrow, intracellular vacuoles; yellow circle, comparison of ribosomal density.

The authors apologize for this error and state that this does not change the scientific conclusions of the article in any way. The original article has been updated.

