# Supplementary material for: Ribosomal Protein L40e Fused With a Ubiquitin Moiety Is Essential for the Vegetative Growth, Morphological Homeostasis, Cell Cycle Progression, and Pathogenicity of Cryptococcus neoformans
Source: Front Microbiol. 2020 Nov 5;11:570269. doi: 10.3389/fmicb.2020.570269 (PMC7674629; doi:10.3389/fmicb.2020.570269)
Supplement: Supplementary file 1 [file Presentation_1.pdf]

Supplemental Materials

Figure S1 Southern hybridization blot. The detailed protocol is described in the Materials and Methods. Lane 1, *ubi1*Δ; Lane 2, *ubi1*Δ*::UBI1*. Genomic DNA from the *ubi1*Δ and *ubi1*Δ*::UBI1* cells was digested with EcoRI, and separated on a 0.8% agarose gel. Before transferring to membranes, the gel of ladder lane was cut, and reutilized to label the DNA marker on the film after hybridization and visualization. The size of the hybridization band is 1720 nt in *ubi1*Δ. The reconstituted strain, *ubi1*Δ*::UBI1*, was constructed by ectopic integration of a reconstituted vector. Therefore, we cannot predict the size of its hybridization band.

Figure S2. The growth rate of *C. neoformans* was positively correlated with its *UBI1* expression levels. A. The P_{CTR4} reconstituted strain displayed different *UBI1* expression levels in copper-limited and copper-rich media. Real-time PCR was performed to test the transcriptional level of *UBI1* under different conditions (1, YPD containing 25 μM $CuSO_4$; 2, YPD; 3, YPD containing 50 μM BCS). Obviously, *UBI1* expression was inversely correlated with the concentration of copper ions in the P_{CTR4}-*UBI1* reconstituted strain. B. The wild-type strain displayed similar growth rates in different media.

Figure S3. Ubi1 was involved in various stress responses of *C. neoformans*. Strains were cultured to saturation at 30 ℃ in liquid YPD medium, 10-fold serially diluted ($1\text{-}10^6$ dilutions), and then 3 μL cells spotted onto YPD or YNB agar (see Experimental procedures). They were incubated under specified conditions for two weeks and photographed.

Table S1. Strains and plasmids used in this study.

Strain & Plasmid	Genotype	Reference
C. neoformans		
H99	*MAT α*	Perfect et al. (1993)
*ubi*Δ	*MAT α ubi1::NEO*	This study
*ubi1*Δ*::UBI1*	*MAT α ubi1::NEO+ UBI1::NAT*	This study
*ubi1*Δ*::RPL40a*	*MAT α ubi1::NEO+ RPL40a::NAT*	This study
P_{CTR4}-*UBI1*	*MAT α +CTR4-UBI4::NEO*	This study
**Plasmid**		
pJAF1	*NEO* resistance gene	O'Meara TR et al.(2010)
pCH233	*NAT* resistance gene	O'Meara TR et al.(2010)
pUBI1-NAT	Modified pCH233 containing gene *UBI1*	This study
pNEO-CTR4	CTR4 promoter and *NEO* resistance gene	This study
pRPL40a-NAT	*UBI1* promoter，*RPL40a* ORF, and *NAT* Resistance gene	This study

Table S2. Primers used in this study.

Primer	Sequence(5'-3')	Function
ZJY001	CAATCACAGCAACAGTCG	Construction of knocking-out or promoter-replacing cassatte
ZJY002	ACTGGCGGCCGTTACTAGTAAAACAAAGATCCTTATCA	Construction of knocking-out or promoter-replacing cassatte
ZJY003	CTGGCCGTCGTTTTACGCCGAGCTAGATGAGTTTTA	Construction of knocking-out cassatte
ZJY004	AAGCGCAACTAGTGAAGAAG	Construction of knocking-out cassatte
ZJY005	TAGTAACGGCCGCCAGT	Construction of knocking-out cassatte
ZJY006	TTAAATCACACCCCAAAGAC	Analysis of proper homologous recombination of *ubi1*Δ
ZJY007	AGATCAGCAAAGAAGTGAGG	Analysis of proper homologous recombination of *ubi1*Δ
ZJY008	CGAGCATGCATCTAGACCTGAAGCGACAACCTG	Reconstitiution for *ubi1*Δ mutant
ZJY009	AATTGGGCCCTCTAGACTGTCTTCCCTCCTCTT	Reconstitiution for *ubi1*Δ mutant
ZJY010	GATTGTTTGCAGTCTTACCG	Analysis of reconstituted strain *ubi1*Δ*::UBI1*
ZJY011	GGAAGTCAAAACATACGCAG	Analysis of reconstituted strain *ubi1*Δ*::UBI1*
ZJY012	CGACAACGACTTCACCAATC ATGCAGATCTTCGTCAAGA	Construction of promoter-replacing cassatte
ZJY013	GAGAACAGCGGAACAACT	Construction of promoter-replacing cassatte
ZJY014	TAGTAACGGCCGCCAGT	Construction of NeoR-PCTR4 fragment
ZJY015	GATTGGTGAAGTCGTTGTCG	Construction of NeoR-PCTR4 fragment
ZJY016	ACCACCTCGGAGACGGA	Construction of fragment containing the *UBI1* promoter and monoubiquitin ORF
ZJY017	CGTCTCCGAGGTGGTTAAACTTGCTTGTATTCTC	Construction of the *UBI1* terminator fragment
ZJY018	CATCTGTAAAACAAAGATCC	Construction of the *UBI1* promotor fragment
ZJY019	CTTTGTTTTACAGATG ATCATCGAGCCCTCTCTC	Consruction of the fragment containing *RPL40a* ORF and the *UBI1* terminator
ZJY020	ACAATGTCAAGTCCAAGATC	Confirmation of P_{CTR4}-*UBI1* mutant via real-time PCR
ZJY021	CACAGTTGTACTTAGAAGCA	Confirmation of P_{CTR4}-*UBI1* mutant via real-time PCR
ZJY022	GGCCCTTGCTTCTAAGTACA	Confirmation of partial reconstituted strain *ubi1*Δ*::RPL40a* via real-time PCR
ZJY023	TCGGATCTGGGAAGAGTG	Confirmation of partial reconstituted strain *ubi1*Δ*::RPL40a* via real-time PCR
M13F	GTAAAACGACGGCCAG	Construction of knocking-out cassatte
NEO-F	TATGTCCTGATAGCGGTCCG	Creation of NEO Probe for Soutern

		analysis
NEO-R	AAGATGGATTGCACGCAGG	Creation of NEO Probe for Soutern analysis
Nat-F	ACCTCTGGCTGGAGGTCAC	Creation of NAT Probe for Soutern analysis
Nat-R	GGGCATGCTCATGTAGAGC	Creation of NAT Probe for Soutern analysis
ACT1-a	AGGTCATCACCATTGGTAACG	Real-time PCR
ACT1-b	CATTGTACATGGTAGTACCAC	Real-time PCR
