# Supplementary figures and images for: Ribosomal Protein L40e Fused With a Ubiquitin Moiety Is Essential for the Vegetative Growth, Morphological Homeostasis, Cell Cycle Progression, and Pathogenicity of Cryptococcus neoformans
Source: Front Microbiol. 2020 Nov 5;11:570269. doi: 10.3389/fmicb.2020.570269 (PMC7674629; doi:10.3389/fmicb.2020.570269)

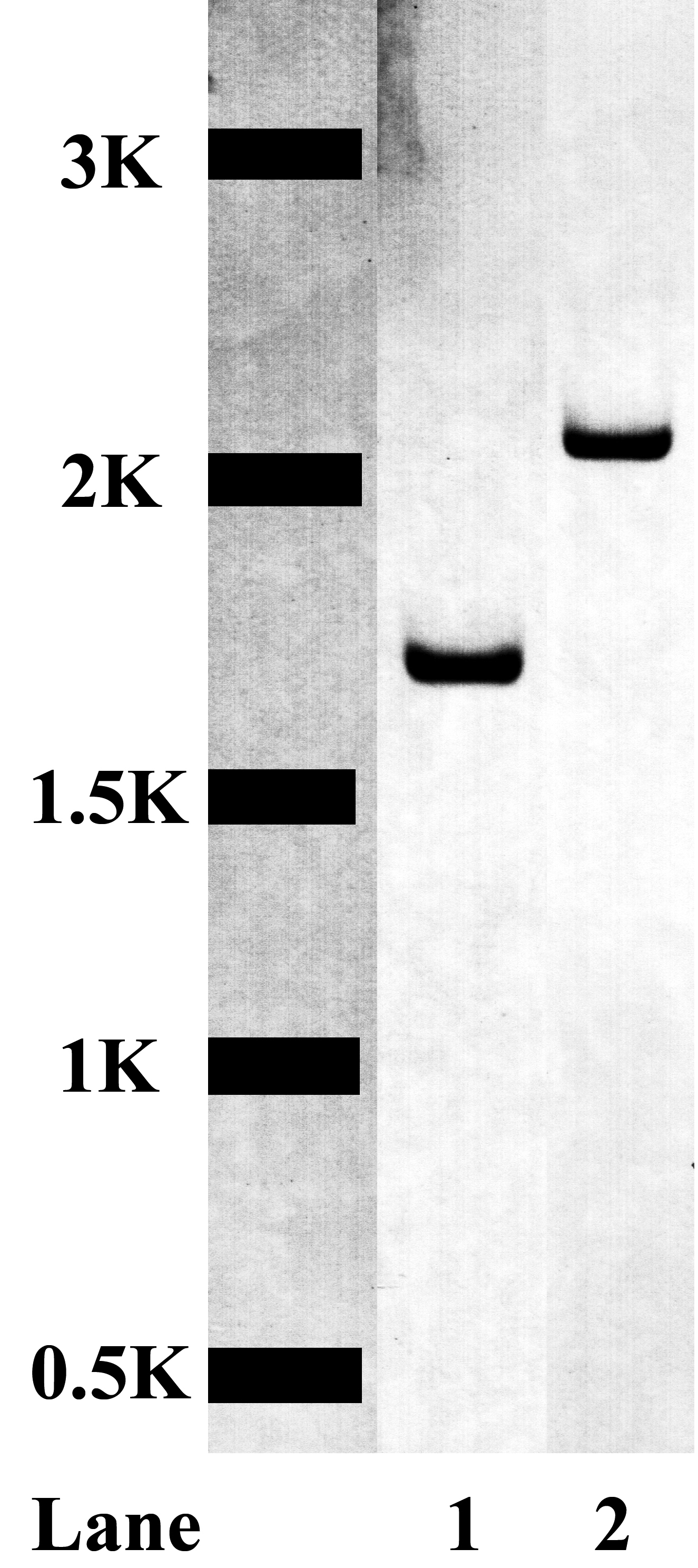

Supplement: Supplementary file 2 [file Image_1.JPEG]

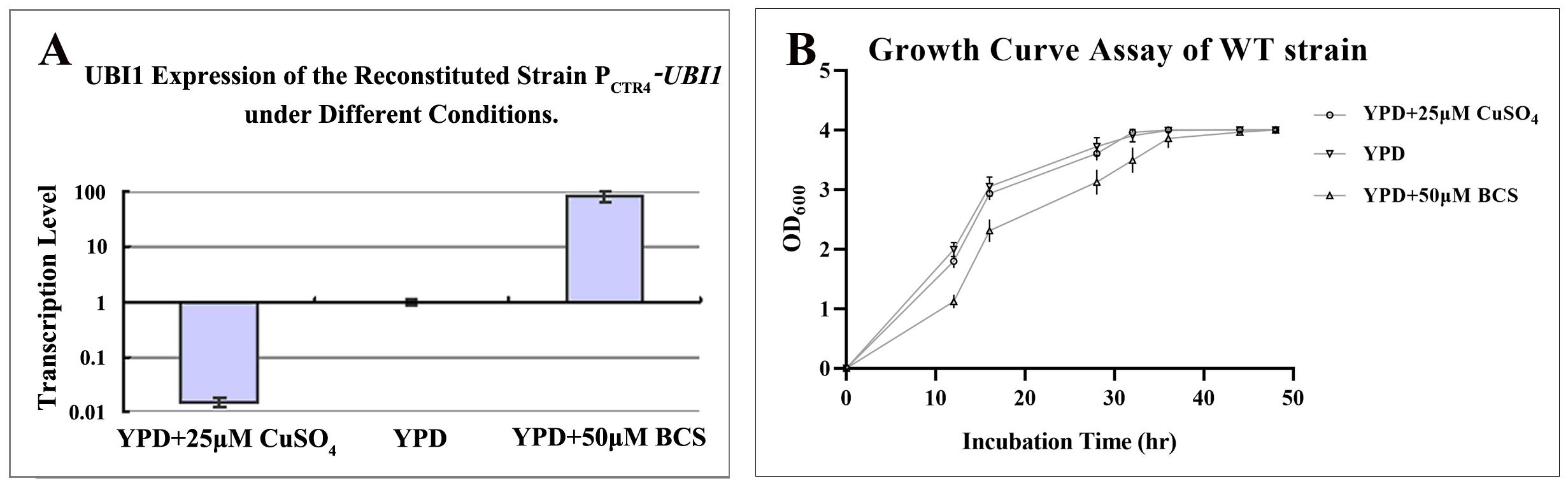

Supplement: Supplementary file 3 [file Image_2.JPEG]

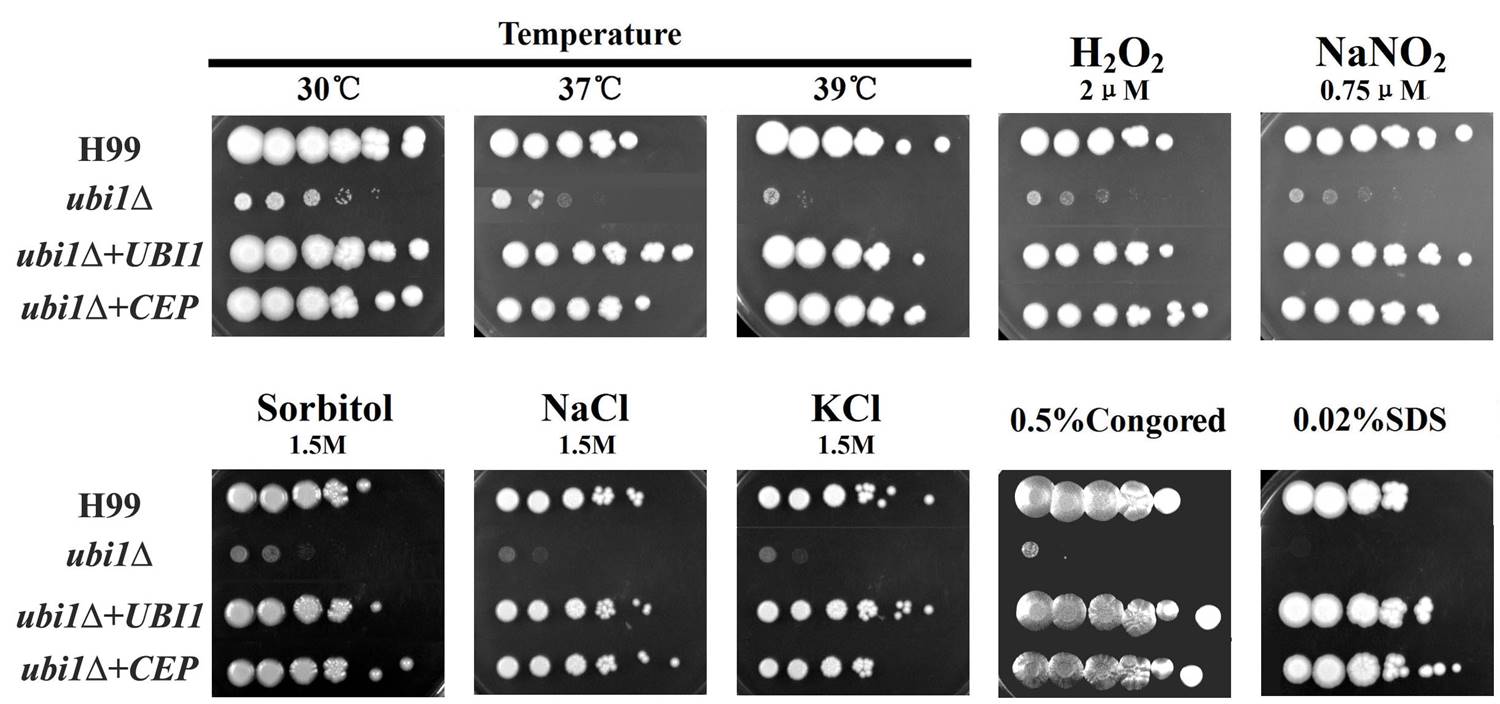

Supplement: Supplementary file 4 [file Image_3.JPEG]
